# Prevalence of metabolic syndrome in polycystic ovarian syndrome women in a hospital of Tehran

**Published:** 2012-03

**Authors:** Ashraf Moini, Fatemeh Javanmard, Bita Eslami, Najmeh Aletaha

**Affiliations:** 1 *Department of Obstetrics and Gynecology, Arash Women’s Hospital, Tehran University of Medical Sciences, Tehran, Iran.*; 2 *Department of Endocrinology and Female Infertility, Royan Institute of Reproductive Biomedicine (ACECR), Tehran, Iran.*; 3 *Department of Internal Medicine, Tehran University of Medical Sciences, Tehran, Iran.*

**Keywords:** *Dyslipidemic*, *Metabolic Syndrome*, *Prevalence*, *Polycystic Ovarian Syndrome*

## Abstract

**Background:** Polycystic ovarian syndrome (PCOS) is a condition associated with chronic anovulation, insulin resistance and androgen excess. Women with this syndrome are at increased risk of metabolic syndrome.

**Objective: **The aim of the present study was to determine the prevalence of metabolic syndrome (MBS) in women with PCOS referred to Arash Hospital in different ages and body mass index (BMI).

**Materials and Methods: **A cross-sectional study was conducted in Gynecologic Clinic at Arash Hospital affiliated with Tehran University. A total of 282 women with PCOS ages between 15-40 years were included. The prevalence of Metabolic Syndrome and its components in this population were the main outcomes. Height, weight, waist circumference, blood pressure and laboratory tests (FBS, TSH, HDL-C, serum prolactin, triglycerides and total cholesterol) were measured in this population.

**Results:** The prevalence of MBS in PCOS women was 22.7% (64 cases). The rate of central obesity, FBS more than 110 mg/dl, triglycerides more than 150 mg/dl, high-density lipoprotein cholesterol levels (HDL-C) less than 50 mg/dl, and blood pressure ≥130/85 mmHg in PCOS women was 31% (87), 3.2% (9), 33% (93), 68.8% (194), and 10.6% (30), respectively. The risk of MBS was increased in older and the obese women (BMI ≥30 kg/m^2^).

**Conclusion:** The present sample showed women with PCOS have a high prevalence of MBS and its individual components, particularly decreased HDL-C.

## Introduction

Polycystic ovarian syndrome (PCOS) is a condition associated with chronic anovulation, insulin resistance and androgen excess. It is considered to be one of the most common endocrinopathies among reproductive age women. It affects approximately 6-10% of reproductive-age women in the United States (1). 

Some clinical manifestations of PCOS are hyperandrogenism, oligomenorrhea or amenorrhea, hirsutism and chronic anovulation. Women with this syndrome are at increased risk of metabolic syndrome (MBS: X syndrome and insulin resistance syndrome). MBS consists of a constellation of metabolic abnormalities that confer increased risk of cardiovascular disease and diabetes mellitus (2). 

“The National Cholesterol Education Program Adult Treatment Panel (NCEPATP III) guidelines define MBS as having three or more of the following abnormalities: 1. waist circumference in females greater than 88 cm, 2. fasting serum glucose at least 110mg/dl, 3. fasting serum triglycerides at least 150mg/dl, 4. serum high density lipoprotein cholesterol (HDL-C) less than 50 mg/dl and 5. blood pressure at least 130/85mmHg” (3). 

Although insulin levels are not used to diagnose either PCOS or MBS, however insulin resistance and compensatory hyperinsulinemia are key pathogenic factors in the pathogenesis of these disorders (2). It seems the prevalence of MBS in PCOS patients is higher than general population. 

United States studies confirmed the prevalence of the MBS in PCOS women (43-46%) was nearly 2-fold higher than that reported for aged-matched women in the general population (4, 5). Two different studies which were conducted in Iran had controversy. 

The first study by Lankarani *et al* manifested the criteria for MBS are frequently present in young women with PCOS and more useful as a prognostic factor than insulin resistance. Meanwhile they suggested the evaluation of insulin resistance in older age women with PCOS (6). 

Another case-control study by Hosseinpanah *et al* showed MBS was no frequent in a sample of PCOS Iranian population than in healthy control (7). It seems evaluation of MBS in PCOS patients needs more investigation in our population. Therefore, the aim of the present study was to determine the prevalence of MBS in women with PCOS referred to Arash Women’s Hospital in different ages and body mass index (BMI).

## Materials and methods

We evaluated 282 women with PCOS who referred to the Gynecologic Clinic at Arash Women’s Hospital affiliated with Tehran University, Iran, from September 2008 to September 2009. The Ethics Institutional Review Board of Tehran University of Medical Sciences approved the study and informed consent was obtained from all participants. 

PCOS patients were diagnosed with using the Rotterdam criteria (two out of three following criteria were sufficient for diagnosis of PCOS): (1) irregular menstruation, (2) clinical and/or biochemical signs of hyperandrogenism and (3) polycystic ovaries (presence of 12 or more follicles in each ovary, 2-9 mm in diameter and/or increased ovarian volume>10mL) (8). 

Irregular menstruation was defined as oligomenorrhea (eight or fewer menstrual periods annually) or amenorrhea (abnormal suppression or absence of menstruation). MBS was diagnosed using the Adult Treatment Panel-III (ATP_III) guidelines when any three of the following were present: central obesity, raised triglycerides ≥150mg/dl, high-density lipoprotein (HDL) cholesterol <50 mg/dl, blood pressure ≥ 130/85 mm Hg and fasting blood glucose (FBS) ≥110 mg/dl (3). 

Exclusion criteria were: PCOS women younger than 15 years old (due to the possibility overlap with other conditions associated with hyperandrogenism such as congenital adrenal hyperplasia), women older than 40 years (to avoid potential overlap with perimenopause), hyperprolactinemia and hypothyroidism (measure of serum prolactin and TSH levels).

Inclusion criteria were: age 15-40 years, no medication use which affected sex hormones from six months prior to study onset (e.g., oral contraceptives and metformin). Information data and reproductive history of all cases were recorded, such as: age, age of menarche, history of pregnancy and infertility. Height, weight, waist and hip circumferences were measured by the nursing staff. Blood pressure was measured twice in either the right or left arms after the patient was seated and at rest for a minimum of 15 min. The systolic and diastolic measurements reported represented the mean of the two readings.

Patients were stratified into the following: group 1:16-20 years, group 2: 21-25 years, group 3: 26-30 years, group 4: 31-35 years and group 5: 36-40 years; and BMI categories <18.5, 18.5-24.9, 25-29.9, and >30. Waist circumference greater than 88 cm was considered as central obesity and was measured at the level of the umbilicus with flexible tape. 

Laboratory tests were performed at the Arash Women’s Hospital laboratory and included: FBS, HDL-C, serum prolactin, TSH, triglycerides and total cholesterol. TSH and prolactin were measured by ELISA. By considering the other studies, we estimated the prevalence of MBS in our population will be about 20% (15-25%) with 5% confidence interval. So, 256 samples would be enough to find the prevalence. 

**Statistical analysis**

Statistical analysis was performed with SPSS software (version 13). Data were presented as mean±standard deviation and percentages (numbers), when appropriate. The comparison between continuous variables was performed with ‘t-test’. A two-tailed p value of less than 0.05 was considered significant. 

## Results

**Prevalence of MBS**

Prevalence of MBS in PCOS women was 22.7% and occurred in 64 out of 282 subjects. The rate of metabolic syndrome components in total PCOS population and women with metabolic syndrome is shown in table I. The most important component in PCOS population was HDL-C less than 50 mg/dl which was manifested in 68.8% of the present sample. Table II shows the frequency of PCOS and MBS in different age categories. 

It was revealed that the risk of MBS increased with age. Meanwhile, the frequency of PCOS and MBS in BMI categories is shown in table III. The risk of MBS increased in the obesity category (BMI ≥30 kg/m^2^), where 41.5% of PCOS patients who had BMI ≥30 kg/m^2^ manifested MBS.

**Characteristics of women with and without MBS**

As shown in table IV, women with MBS were significantly older than others (p<0.001). As expected, compared with women who did not meet the criteria for MBS, those with MBS had a significantly higher BMI and waist to hip ratio. 

**Table I:** The rate of metabolic syndrome components in total PCOS population and metabolic syndrome women

	**PCOS (N=282)**	**MBS (N=64)**
Central obesity	31% (87)	60.9% (39)
FBS more than 110 mg/dl	3.2% (9)	10.9% (7)
Triglycerides more than 150 mg/dl	33% (93)	81.3% (52)
HDL-C less than 50 mg/dl	68.8% (194)	96.9% (62)
Blood pressure ≥ 130/85 mm Hg	10.6% (30)	34.4% (22)

Data are presented as a percentage with number in parenthesis.

**Table II:** The frequency of PCOS and MBS in different age categories

	**16-20 y**	**21-30 y**	**31-40 y**
*PCOS	6 (2.1)	192 (68.1)	84 (29.8)
**MBS	0/6 (0)	36/192 (18.8)	28/84 (33.3)

* Data are presented as numbers (percentages).

** Percentages in MBS refer to percentages within age categories.

**Table III:** The frequency of PCOS and MBS in different BMI categories

	**<18.5**	**18.5-24.9**	**25-29.9**	**≥30**
*PCOS	6 (2.1)	73 (25.9)	121 (42.9)	82 (29.1)
**MBS	0/6 (0)	4/73 (5.5)	26/121 (21.5)	34/82 (41.5)

* Data are presented as numbers (percentages).

** Percentages in MBS refer to percentages within BMI categories.

**Table IV:** Comparison of PCOS women with and without metabolic syndrome

**Variables**	**Without MBS** **(N= 218)**	**With MBS** **(N=64)**	**p-value**
Age	27.98 ± 4.35	30.31 ± 4.09	<0.0001
BMI	27.03 ± 4.72	31.35 ± 4.83	<0.0001

Data are mean±standard deviation.

## Discussion

The results of our study show the frequency of MBS in reproductive age women with PCOS to be 22.7% which is similar to the prevalence of MBS in other ethnicities and races diagnosed with PCOS (9, 10). However in some ethnic groups, such as the American population, the prevalence is higher than our study, at about 40% (4, 5). These differences are possibly related to factors such as age, diet and lifestyle that cause increased waist circumferences, hypertriglyceridemia and reduced HDL cholesterol levels as important components for MBS.

Azizi *et al* study which was performed in an urban population of Tehran has shown the prevalence of MBS in women was more commonly seen than in men and it was estimated to be 26.1% at ages 20-39 years old (11). However, in our study the prevalence of MBS in PCOS patients (small population) was near (22.7%) to this study in a large and general population. Therefore it seemed that the frequency of MBS in PCOS women was higher than the total population of women. 

The results of a case-control study in Iranian population manifested, the criteria of MBS was frequently higher in young women with PCOS (6). However this result was not confirmed by another study that was suggested more consideration about the association between PCOS and MBS (7). Although in second study the prevalence of insulin resistance in PCOS appeared to be higher than in controls (7). It should be mentioned the differences between these two studies maybe due to different diagnosis criteria for MBS and sample size. 

In the present study the rate of MBS increased by age with a markedly higher prevalence than the 6.7% MBS prevalence reported in women between the ages of 20-30 years and the 15% prevalence reported in women ages 30-40 years from the Third National Health and Nutrition Examination Survey (NHANES III) (12).

The pathogenesis of MBS and its components are complex and not well understood, although insulin resistance and perhaps hyperinsulinemia are considered to be key pathogenic factors in the development of other components of MBS such as abnormal glucose tolerance, dyslipidemia and hypertension (2).

Based on the results of the present study, the high rate of HDL-C < 50 mg/dl (96.9% of cases) with MBS, and triglycerides ≥ 150 mg/dl (81.3% of cases) manifested a significant abnormal lipid profile in PCOS women. Many studies have shown low HDL-C levels, which is one component of the lipid profile that indicates which MBS has an important role in the increasing risk of cardiovascular and coronary artery disease (13-15). Therefore it is appropriate to be concerned when HDL-C levels are less than 50 mg/dl, particularly in high risk populations. Two important factors in the increasing rate of MBS are age and BMI. By categorization of patients by age and BMI, we found increased MBS with age and BMI (Tables I, II). 

Thus as described above, we propose that the criteria of MBS especially HDL-C in all patients with PCOS must be evaluated, particularly in elder and obese patients. It must be mentioned that since the samples were collected from only one hospital in Tehran (capital city of Iran) so the resulted prevalence may not be generalized and further study with more samples from other parts of Iran and another type of study is required. 

In conclusion, the present study indicates that women with PCOS have a high prevalence of MBS and its individual components, particularly decreased HDL cholesterol levels. Therefore the management of these women as a high risk population for MBS is recommended.
